# Cardiovascular inflammation in healthy women: multilevel associations with state-level prosperity, productivity and income inequality

**DOI:** 10.1186/1471-2458-12-211

**Published:** 2012-03-20

**Authors:** Cheryl R Clark, Paul M Ridker, Mark J Ommerborn, Carrie E Huisingh, Brent Coull, Julie E Buring, Lisa F Berkman

**Affiliations:** 1 Division of General Medicine and Primary Care, Brigham and Women's-Faulkner Hospitalist Program, Harvard Medical School, Boston, Massachusetts, USA; 2 Center for Community Health and Health Equity, Brigham and Women's Hospital, Boston, Massachusetts, USA; 3 Division of Preventive Medicine, Brigham and Women's Hospital, Boston, Massachusetts, USA; 4 Center for Cardiovascular Disease Prevention, Brigham and Women's Hospital, Boston, Massachusetts, USA; 5 Division of Cardiovascular Diseases, Brigham and Women's Hospital, Harvard Medical School, Boston, Massachusetts, USA; 6 Department of Biostatistics, Harvard School of Public Health, Boston, Massachusetts, USA; 7 Harvard Center for Population and Development Studies, Harvard School of Public Health, Cambridge, Massachusetts, USA; 8 Department of Society, Human Development and Health, Harvard School of Public Health, Boston, Massachusetts, USA

**Keywords:** C-reactive protein, Geography, Income, Inflammation, Multilevel analysis, Poverty, Socioeconomic factors, Women's health

## Abstract

**Background:**

Cardiovascular inflammation is a key contributor to the development of atherosclerosis and the prediction of cardiovascular events among healthy women. An emerging literature suggests biomarkers of inflammation vary by geography of residence at the state-level, and are associated with individual-level socioeconomic status. Associations between cardiovascular inflammation and state-level socioeconomic conditions have not been evaluated. The study objective is to estimate whether there are independent associations between state-level socioeconomic conditions and individual-level biomarkers of inflammation, in excess of individual-level income and clinical covariates among healthy women.

**Methods:**

The authors examined cross-sectional multilevel associations among state-level socioeconomic conditions, individual-level income, and biomarkers of inflammation among women (n = 26,029) in the Women's Health Study, a nation-wide cohort of healthy women free of cardiovascular diseases at enrollment. High sensitivity C-reactive protein (hsCRP), soluble intercellular adhesion molecule-1 (sICAM-1) and fibrinogen were measured between 1993 and 1996. Biomarker levels were examined among women within quartiles of state-level socioeconomic conditions and within categories of individual-level income.

**Results:**

The authors found that favorable state-level socioeconomic conditions were correlated with lower hsCRP, in excess of individual-level income (e.g. state-level real per capital gross domestic product fixed effect standardized Βeta coefficient [Std B] -0.03, 95% CI -0.05, -0.004). Individual-level income was more closely associated with sICAM-1 (Std B -0.04, 95% CI -0.06, -0.03) and fibrinogen (Std B -0.05, 95% CI -0.06, -0.03) than state-level conditions.

**Conclusions:**

We found associations between state-level socioeconomic conditions and hsCRP among healthy women. Personal household income was more closely associated with sICAM-1 and fibrinogen than state-level socioeconomic conditions. Additional research should examine these associations in other cohorts, and investigate what more-advantaged states do differently than less-advantaged states that may influence levels of cardiovascular inflammation among healthy women.

## Background

Cardiovascular inflammation is a major contributor to atherosclerosis-related cardiovascular disease [1,2]. Biomarkers of cardiovascular inflammation, including acute phase reactants such as high-sensitivity C-reactive protein (hsCRP) and fibrinogen, and markers of vascular wall inflammation such as soluble intercellular adhesion molecule-1 (sICAM-1), each predict the risk of future cardiovascular events, including incident myocardial infarction and stroke, particularly among women [3,4]. Processes of inflammation reflect dynamic interactions among cardiovascular disease risk factors including cholesterol levels and high blood pressure, metabolic conditions including obesity, insulin resistance and diabetes, and behavioral factors such as smoking and exercise [1,5-7].

An emerging literature also suggests that processes of inflammation are socially patterned in healthy women. In the United States, biomarkers of cardiovascular inflammation have been associated with the socioeconomic status (SES) of individuals, and the SES of small geographic areas, namely residential neighborhoods [8,9]. However, the macro socioeconomic conditions of larger geographic areas have yet to be explored in relation to inflammation among healthy women. The so-called "macrosocial," or macro socioeconomic determinants of health are thought to represent the large-scale distribution of resources and policies that impact population health [10]. It is known that traditional cardiovascular disease risk factors and outcomes, including hypertension, diabetes and stroke, show variation at large regional and geographic scales in the United States [11-14]. We have recently shown that there is state-level geographic variation in inflammatory biomarkers among healthy women that is not completely explained by clinical and behavioral characteristics [15]. Despite the recognition of patterns of risk in large geographic areas, few data explore macro socioeconomic characteristics as contributors to these outcomes. Recent data from the REGARDS study show that women who reside in states in the southeastern US - known as the "stroke belt"- have high levels of inflammation, measured by hsCRP [12]. However, in the REGARDS study, socioeconomic status measured at the individual level did not fully explain the effect of state residence on the risk of inflammation among women [12].

Macro socioeconomic conditions measured at the state-level have not been evaluated as correlates of cardiovascular inflammation among women. Our study investigated the association between state-level macro socioeconomic conditions and individual-level biomarkers of inflammation among healthy women free of cardiovascular diseases in the Women's Health Study. Specifically, we hypothesized that state-level socioeconomic conditions, namely, state-level wealth and prosperity, labor productivity, economic growth, poverty and income inequality would be associated with biomarkers of inflammation, and that these associations would not be completely explained by individual-level household income and clinical conditions among healthy women. Based on prior work by Diez-Roux et al, we additionally hypothesized that variability in biomarker levels would depend on both state-level socioeconomic conditions as well as personal household income [16]. We assess the relative contributions of individual-level and state-level socioeconomic conditions in a multilevel analytic framework.

## Methods

### Study population

The present study is a cross-sectional analysis conducted with baseline data from the participants of the Women's Health Study (WHS). The WHS is a nation-wide randomized-controlled trial of the efficacy of aspirin and vitamin E in the prevention of cardiovascular disease and cancer among women [17,18]. Methods for participant recruitment have been described previously [19]. The baseline WHS cohort consists of 39,876 healthy middle-aged and elderly women aged 38 and older who were without cardiovascular disease or cancer at study entry between 1993 and 1996. At study entry, all participants completed questionnaires to survey their baseline demographic, clinical, and lifestyle/behavioral characteristics. Baseline blood samples were obtained from 28,296 participants to quantify levels of traditional and novel cardiovascular risk factors [18,20]. The Partners Instituional Review Board reviewed and approved this study.

### Analytic sample

Our initial sample consisted of all 28,296 partcipants with a baseline blood sample. Data were missing in 12.8% of the study cohort (n = 3,632). The largest source of missing data was item non-response due to missing survey data on the personal annual household income of WHS participants (n = 1,504 accounting for 5.3% of the data). Missing data on income were correlated with participant's age, cholesterol levels, body mass index and hsCRP, where those missing income data tended to be older, have lower total cholesterol levels, lower body mass index and lower hsCRP. Thus we imputed missing data for income in multivarible models, using procedures for multiple imputation [21] in SAS^® ^version 9.2 (SAS Institute, Cary, NC) with the PROC MI and PROC MIANALYZE procedures.

Non-imputed samples (n = 24,664) excluding the 3,632 participants with missing income data were used in descriptive analyses. The final analytic cohort used in multivarible analysis consisted of 26,029 participants with complete data after imputing missing data on income.

### Measures

#### Outcome measures: biomarkers of cardiovascular inflammation

The study outcome measures were biomarkers of inflammation as quantified by blood plasma levels of (1) hsCRP (mg/L), (2) sICAM-1 (ng/ml) and (3) fibrinogen (mg/dL), each assessed separately. Assays used to quantify biomarker levels have been described [20].

#### Key predictor variables: state-level macro socioeconomic conditions

The primary predictor variables were measures of state-level socioeconomic conditions: (1) state-level *wealth and prosperity*, (2) state-level *labor productivity*, (3) state-level *poverty*, (4) state-level *income inequality *and (5) state-level *average annual economic growth*. State-level *wealth and prosperity *were assessed with two separate measures (a) 1990 state-level real per-capita gross domestic product (GDP) from the Bureau of Economic Analysis, and (b) the 1990 US Census state-level median household income. GDP at the state-level in 1990 was calculated by the Bureau of Economic Analysis using the Office of Management and Budget Standard Industrial Classification (SIC) [22]. GDP data were computed from all industry activity within that year, accounting for inflation ("real GDP"), and scaled for state population ("per capita GDP") to allow comparison across states of different sizes [22,23]. Median household income was taken from the 1990 US Decennial Census, and represents money income received in the 1989 calendar year from related and non-related household members aged 15 years and over. The US Census estimated median income at the state-level as calcuated from wages and salary income, self-employment income, interest income, dividends, rental and royalty income, and money income from social security, public assistance and welfare income [24].

State-level *labor productivity *describes the value of workers' output - i.e., what workers do - as a contribution to the wealth of the economy, [25,26] in contrast to wealth gained through capital income [25,27]. State per employee earnings has been suggested as a measure of labor productivity as it captures both the value of goods and services produced by workers, as well as the resources that accrue back to the employed population through wages and salaries [25,28,29]. Per employee earnings were calculated by the Morrison Institute for Public Policy using Bureau of Economic Analysis data on earnings from wages and salaries, proprietors' income, employer contributions to employee pensions and insurance payments, as distributed across the employed population of the region. We obtained state-level data on per employee earnings from the PEW Center on the States for this analysis [25,26,29].

*Poverty *at the state-level was obtained from the 1990 US Decennial Census long form survey, measured as the percentage of the total state-population with an annual household income under the 1989 federal poverty threshold, accounting for household size and age of the householder. State-level *income inequality *was measured as the Gini index of inequality. State-level Gini coefficients were obtained from the 1990 Decennial Census based on 1989 household income data from the Census long form survey [30].

The average annual growth in state-level real per capita GDP was used as the measure of *economic growth *at the state-level. Average annual growth statistics were calculated by the Bureau of Economic Analysis across the decade for which inflammatory biomarker data were collected in the study, between 1990 and 1996.

##### Individual-level covariates: cardiovascular risk factors and personal annual household income

Individual-level covariates thought to correlate with inflammation included in the analysis were: age, race/ethnicity (non-Hispanic White versus non-White race/ethnicity), body mass index (normal weight versus overweight and obese body mass index), low-density lipoprotein cholesterol (LDL-C), high-density lipoprotein cholesterol (HDL-C), systolic blood pressure category, the presence of diabetes (defined by participant self-report and measured blood hemoglobin A1C equal or greater than 6.5%), frequency of exercise (recreational physical activity performed rarely or never, less than once per week, 1 to 3 times per week, four or more times per week), average daily caloric intake, smoking status (never smoked, prior smoker, current smoker), and the personal annual household income of the participant. Assays used to quantify the blood-derived measures were certified by the National Heart, Lung, and Blood Institute/Centers for Disease Control and Prevention Lipid Standardization Program [20].

### Statistical analysis

#### Descriptive data

We present descriptive means, medians and percentages of demographic, behavioral and clinical characteriscs of WHS participants by state-level median household income, as well as ranges of all the state-level socioeconomic measures. We used Spearman rank correlation coefficients calculated in SAS^® ^to assess correlations among state-level socioeconomic measures.

#### Multilevel associations with personal income and state-level socioeconomic conditions

We hypothesized that biomarkers levels would vary depending on both state-level socioeconomic conditions as well as the participant's personal household income [16]. Thus, we present figures describing the median values of biomarkers of inflammation within quartiles of state-level socioeconomic conditions, and across categories of personal household income. To test for a multilevel effect of state-level socioeconomic conditions on inflammatory biomarker levels in excess of personal household income, we used the PROC MIXED procedure in SAS^® ^to estimate associations between biomarkers of inflammation and state-level measures, adjusted for individual-level personal income and covariates. Standardized beta coefficient fixed effect estimates with 95% confidence intervals are reported from multilevel models. Due to the known skew toward lower values, hsCRP was log-transformed prior to statistical analysis.

#### Adjustment for covariates and handing of missing data in multivariable models

In multivariable models, we used a propensity score predicting body mass index (normal weight versus overweight and obese status) to account for the causal relationships between adiposity and several metabolic and behavioral variables (exercise, caloric intake, HDL-C, LDL-C, diabetes, systolic blood pressure) as they relate to inflammation [31]. We report multivariable analyses with imputed data for income. Sensitivity analyses in multivariable multilevel models showed that effect estimates were not substantively different in models with and without missing data on income.

## Results

Table 1 describes demographic, behavioral and clinical characteristics of the WHS study cohort by quartiles of state-level median household income. State-level distribution of participants in the Women's Health Study have been detailed previously [15]. WHS participants who lived in states in the highest quartile of state median income (e.g., California, Connecticut, Massachusetts) tended to be younger, had higher personal incomes, less frequently smoked, more frequently exercised, were less likely to be obese, were less likely to have diabetes, and had a better HDL-C profile than WHS participants living in states in the lowest quartile of state median income (e.g., Mississippi, West Virginia, Arkansas). High-income states had a higher percentage of non-White WHS participants, chiefly Asian/Pacific Islander groups (e.g., California, 10% non-White WHS participants) than lower-income states (e.g., West Virginia, 2% non-White WHS participants). Caloric intake was slightly higher among WHS participants in the highest-income states than lower-income states. The mean systolic blood pressure category was not substantively different across quartiles of state-level median household income. The median value of each inflammatory biomarker decreased with increasing quartiles of state-level median household income (Table 1).

**Table 1:** Selected Demographic, Lifestyle, and Clinical Characteristics of Women's Health Study Participants: By Quartiles of State-Level Median Household Income

		Quartiles of State-Level Median Household Income ^a,b^			
	**WHS Participants ^c^** **N = 24,664**	**$20,136-$24,807**	**$25,257-$27,854**	**$28,706-$31,183**	**$32,181-$41,721**

**Demographic**					

Age, median (IQR), y	53 (49-59)	53 ± 0.1	53 ± 0.1	53 ± 0.1	52 ± 0.1

NH-White ^d^	23,419 (95)	95	95	97	93

Non-White	1,245 (5)	5	5	3	7

Hispanic	216 (1)	1.0	1.3	0.4	1.0

Non-Hispanic Black	422 (2)	2.2	1.5	1.1	2.3

Asian/Pacific Islander	327 (1)	0.5	0.9	0.8	2.4

Other/unknown race/ethnicity	280 (1)	1.3	1.1	0.9	1.3

Lowest income (< $19,999)	1,207 (4.9)	6.6	5.6	5.1	3.5

2 ($20,000-29,999)	2,379 (9.7)	11.8	11.3	10.4	6.8

3 ($30,000-39,999)	3,426 (13.9)	15.8	16.0	14.9	10.5

4 ($40,000-49,999)	4,091 (16.6)	18.9	17.8	16.9	14.5

5 ($50,000-99,999)	10,303 (41.8)	36.8	39.3	41.5	45.8

Highest income ($100,000+)	3,258 (13.2)	10.1	10.0	11.1	18.9

**Lifestyle**					

Current smoker	2,848 (12)	13	12	11	11

Exercise rarely/never	9,121 (37)	42	37	36	36

Median Daily Caloric intake, median (IQR), kCal	1675 (1350-2053)	1673 ± 10	1660 ± 6	1680 ± 6	1682 ± 6

**Clinical Covariates**					

HDL-C, median (IQR), mg/dL	52 (43-62)	50 ± 0.3	51 ± 0.2	52 ± 0.2	53 ± 0.2

LDL-C, median (IQR), mg/dL	121 (100-144)	122 ± 0.7	121 ± 0.4	121 ± 0.4	121 ± 0.4

BMI, median (IQR), kg/m^2 ^	24.9 (22.5-28.3)	25.1 ± 0.1	24.8 ± 0.1	25.1 ± 0.1	24.6 ± 0.1

Systolic blood pressure category, mean ± SD, mmHg	124 ± 14	124 ± 14	123 ± 14	124 ± 14	123 ± 14

Percent with diabetes	737 (3)	4	3	3	3

**Clinical Outcome Variables**					

hsCRP, median (IQR), mg/L	2.0 (0.8-4.4)	2.4 ± 0.1	2.2 ± 0.1	2.0 ± 0.1	1.8 ± 0.1

sICAM, median (IQR), ng/ml	343 (301-394)	351 ± 2	343 ± 1	343 ± 1	338 ± 1

Fibrinogen, median (IQR), mg/dL	351 (307-403)	355 ± 2	351 ± 1	350 ± 1	349 ± 1

Abbreviations: BMI, Body Mass Index; IQR, inter-quartile range.

^a ^Median household income measured from 1990 U.S. Decennial Census. State-level quartiles are based on ranges of the 50 United States plus the District of Columbia.

^b ^Values are median ± standard error or mean ± standard deviation when appropriate.

^c ^Figures are No.(%) unless otherwise noted.

^d ^"Non-White" includes Black, Asian/Pacific Islander, and American Indian/Native American/Alaska Native.

Table 2 describes the range of socioeconomic conditions at the state level. Spearman rank correlation coefficients show that the measures of wealth and prosperity (real per-capita GDP, state median household income) and labor productivity (per employee earnings) were tightly correlated (Table 3). Wealthy and prosperous states tended to have stable economies (low levels of average economic growth) between 1990 and 1996. Poorer states, measured by the prevalence of state-level poverty, tended to have higher levels of average annual economic growth during this period. State-level income inequality was directly correlated with state-level poverty, and inversely related to median household income (Table 3).

**Table 2:** U.S. State-Level Socioeconomic Conditions: Wealth and Prosperity, Growth, Income Inequality, Poverty and Labor Productivity Measures

	U.S. State-Level Percentile Ranges ^a^ N = 51				
	**5^th^**	**25^th^**	**50^th^**	**75^th^**	**95^th^**

**Wealth and Prosperity**					

Real Per-capita GDP 1990, $	18,872	21,520	24,387	28,369	38,663

Median Household Income 1990, $	21,147	25,257	28,706	32,181	40,927

**Economic Growth**					

Average Annual Growth Real Per-capita GDP 1990-1996	0.17	1.08	2.11	2.94	3.66

**Income Inequality**					

Gini Coefficient of Inequality 1990	0.394	0.412	0.428	0.446	0.475

**Poverty**					

Percent in Poverty 1990 (State-population percentage)	7.6	10.3	12.4	15.7	20.6

**Labor Productivity**					

Per employee earnings 1990, $ ^b^	29,124	32,545	34,502	38,582	47,262

^a ^State-level percentiles are based on ranges of the 50 United States plus the District of Columbia.

^b ^Per employee earnings are not calculated for the District of Columbia.

**Table 3:** Spearman Correlation Coefficients Among State-Level Wealth and Prosperity, Growth, Income Inequality, Poverty, and Labor Productivity

	Wealth and Prosperity		Economic Growth	Income Inequality	Poverty	Labor Productivity
	**Real per-capita GDP ^a^**	**Median Household Income ^a^**	**Average Annual Growth Real Per-Capita GDP 1990-1996 ^a^**	**Gini Coefficient ^a^**	**Percent in Poverty ^a^**	**Per Employee** **Earnings ^a,b^**

**Wealth and Prosperity**						
Real per-capita GDP ^a^	1.00	0.823**	-0.694**	-0.124	-0.616**	0.832**
Median Household Income ^a^	0.823**	1.00	-0.573**	-0.329*	-0.824**	0.821**
**Economic Growth**						
Average Annual Growth Real Per-Capita GDP 1990-1996 ^a^	-0.694**	-0.573**	1.00	0.003	0.391**	-0.591**
**Income Inequality**						
Gini Coefficient ^a^	-0.124	-0.329*	0.003	1.00	0.671**	0.088
**Poverty**						
Percent in Poverty ^a^	-0.616**	-0.824**	0.391**	0.671**	1.00	-0.535**
**Labor Productivity**						
Per Employee Earnings ^a,b^	0.832**	0.821**	-0.591**	0.088	-0.535**	1.00

**Indicates results are statistically significant at *P *< 0.01.

*Indicates results are statistically significant at *P *< 0.05.

^a ^Spearman correlation coefficients among linear variables obtained via Proc Corr procedure in SAS^®^.

^b ^Per employee earnings are not calculated for the District of Columbia.

**Figure 1:**
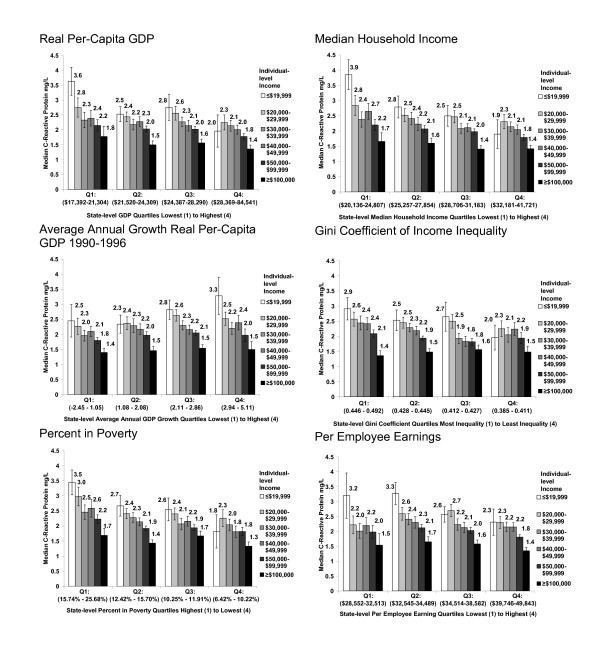
**Median C-reactive protein (mg/L) levels among Women's Health Study participants by state-level characteristics and individual-level household income**. Figures display unadjusted inflammatory biomarker median values and associated standard errors by personal household income within quartiles of state-level characteristics. State-level socio economic conditions are based on ranges of the 50 United States plus the District of Columbia. Per employee earnings are not calculated for the District of Columbia.

Figure 1 shows the multilevel relationship between hsCRP, personal household income, and state-level economic conditions. Across almost all state-level indicators, women with low personal household incomes (≤ $19,999 annually) who lived in states with the *most *favorable economic conditions (wealthy and prosperous, high productivity, low poverty, low inequality) had lower levels of inflammation than similarly low-income women (≤ $19,999 annually) who lived in states with the *least *favorable economic conditions. For example, the median hsCRP for the lowest-income women who lived in states in the least wealthy GDP quartile was 3.6 mg/L (standard error 0.47), compared to 2.0 mg/L (standard error 0.54) for the lowest-income women who lived in states in the most wealthy GDP quartile (Figure 1).

Additionally, for most state-level indicators, the disparity in hsCRP levels between women with the highest ($100,000 and greater) and lowest (≤ $19,999) personal incomes was smallest under the most favorable economic conditions. In particular, state-level income inequality appeared to influence the range of inflammatory hsCRP values. The difference in hsCRP between the highest-income women (median hsCRP 1.5, standard error 0.19), and the lowest-income women (median hsCRP 2.0, standard error 0.41) was 0.5 points among women living in states with the lowest income inequality. The difference in hsCRP between women with the highest and lowest personal income was 1.5 points among women in states with the highest income inequality (Figure 1).

The trend in biomarker values associated with average annual economic growth was the reverse of that seen with other indicators. Women in states with the highest average annual growth tended to have the highest levels of hsCRP, with high hsCRP levels among the lowest-income women.

**Figure 2:**
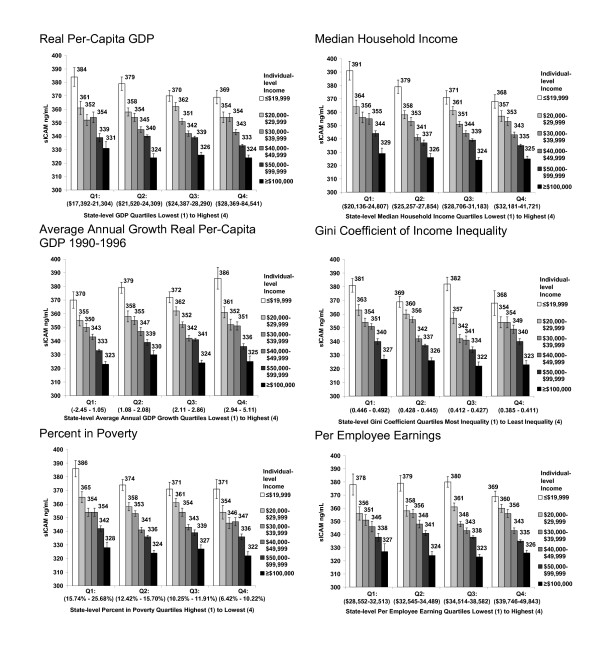
**Median sICAM-1 (ng/ml) levels among Women's Health Study participants by state-level characteristics and individual-level household income**. Figures display unadjusted inflammatory biomarker median values and associated standard errors by personal household income within quartiles of state characteristics. State-level socio-economic conditions are based on ranges of the 50 United States plus the District of Columbia. Per employee earnings are not calculated for the District of Columbia.

**Figure 3:**
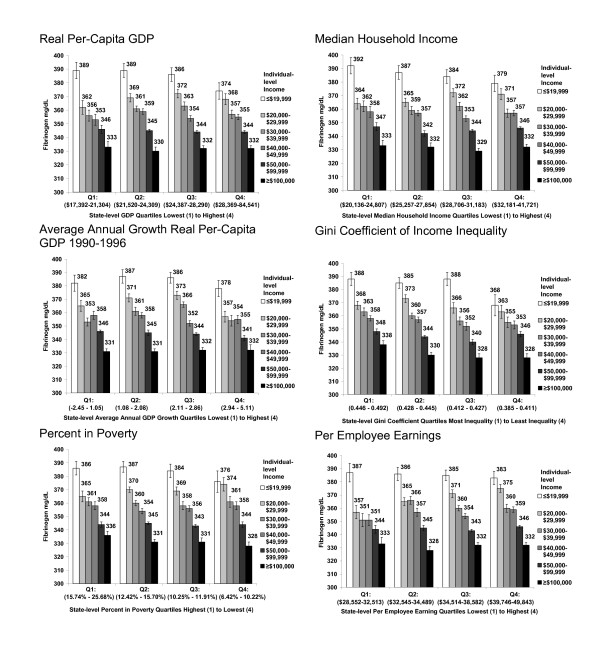
**Median fibrinogen (mg/dL) levels among Women's Health Study participants by state-level characteristics and individual-level household income**. Figures display unadjusted inflammatory biomarker median values and associated standard errors by personal household income within quartiles of state-level characteristics. State -level socioeconomic conditions are based on ranges of the 50 United States plus the District of Columbia. Per employee earnings are not calculated for the District of Columbia.

Figures 2 and 3 display patterns for sICAM and fibrinogen. The protective pattern of favorable state-level economic conditions on sICAM and fibrinogen levels among low-income women was not as pronounced as that seen for hsCRP.

Table 4 quantifies the association between personal household income and biomarkers of inflammation, as well as the association between each state-level characteristic and biomarker of inflammation in excess of personal household income, all adjusted for women's demographic, behavioral and clinical characteristics. Personal household income was independently associated with sICAM-1 and fibinogen, where higher levels of personal income were associated with lower levels of sICAM-1 and fibrinogen, after adjustement for individual-level covariates (Table 4). When adjusted for individual-level covariates, personal household income had a positive association with hsCRP, where higher personal incomes were associated with higher hsCRP. After propensity score adjustment for metabolic variables, this association crossed zero (Std B 0.01, 95% CI -0.005, 0.02).

**Table 4:** Fixed Effect Estimates of Personal Household Income and State-Level SES Measures on Log(hsCRP), ICAM, and Fibrinogen

Multivariable models ^a^	(Log)hsCRP		sICAM-1		Fibrinogen	
	**Effect **	**95% CI**	**Effect**	**95% CI**	**Effect**	**95% CI**
**No state-level covariates**						
Model I. Personal household income category of WHS participant ^b,d^	0.01	-0.005, 0.02	-0.04	-0.06, -0.03**	-0.05	-0.06, -0.03**
**Wealth and Prosperity**						
Model II. State real per-capita GDP quartiles ^b,c,d^	-0.03	-0.05, -0.004*	-0.02	-0.03, -0.002*	-0.001	-0.02, 0.02
Model III. State median household income quartiles ^b,c,d^	-0.05	-0.07, -0.03**	-0.02	-0.03, -0.004*	-0.005	-0.02, 0.01
**Economic Growth**						
Model IV. State annual growth real per-capita GDP quartiles 1990-1996 ^b,c,d^	0.01	-0.01, 0.04	0.01	-0.005, 0.03	-0.02	-0.04, -0.001*
**Income Inequality**						
Model V. State Gini coefficient quartiles ^b,c,d^	0.04	0.01, 0.06**	0.02	0.001, 0.03*	0.03	0.02, 0.05**
**Poverty**						
Model VI. State percent poverty quartiles ^b,c,d^	0.05	0.03, 0.07**	0.02	0.002, 0.03*	0.01	-0.003, 0.03
**Labor Productivity**						
Model VII. State earnings per employee quartiles ^b,c,d^	-0.02	-0.05, -0.001*	-0.01	-0.02, 0.01	0.01	-0.01, 0.03

^a ^All models adjust for age, race and ethnicity, obese and overweight status, smoking status, personal household income category, listed covariates and propensity score predicting obese and overweight status vs. normal weight status as a function of exercise level, average daily caloric intake, HDL, LDL, diabetes status and systolic blood pressure.

^b ^Figures are standardized fixed effect estimates obtained via the Proc Mixed procedure in SAS^® ^version 9.2. Effects are standardized beta coefficients representing the standard deviation change in (Log)hsCRP, sICAM-1, or fibrinogen associated with a 1 standard deviation increase in the listed predictor variable.

^c ^Multi-level models II - VII include state-level data on the 50 United States plus the District of Columbia. State earnings per employee are not calculated for the District of Columbia.

^d ^Missing income values in all multivariable models are imputed using the multiple imputation procedures on the above criteria via Proc MI and Proc MIANALYZE in SAS^® ^version 9.2.

**Indicates results are statistically significant at *P *< 0.01.

*Indicates results are statistically significant at *P *< 0.05.

Income inequality was independently associated with all three biomarkers, where rising quartiles of inequality were associated with rising levels of hsCRP, sICAM-1 and fibrinogen (Table 4).

Rising quartiles of state-level wealth and prosperity (real per-capita GDP and median household income) were independently associated with lower levels of hsCRP and sICAM-1, in excess of personal household income and individual-level covariates (Table 4). Rising quartiles of state-level poverty were associated with an increase in hsCRP and sICAM-1, independent of personal household income. Labor productivity was associated with hsCRP, but not sICAM-1 or fibrinogen. Quartiles of average annual economic growth were independently associated with fibrinogen, but not other biomarkers. The relation between economic growth and fibrinogen levels appeared non-linear; a quadratic term did not appear to fit the data (Std B -0.01, 95% CI -0.03, 0.01).

## Discussion

Our study found that state-level macro socioeconomic conditions were associated with biomarkers of inflammation among healthy women in the Women's Health Study. C-reactive protein appeared to be most sensitive to variation in state-level socioeconomic conditions. High levels of wealth, prosperity and labor productivity, and low levels of state-level poverty and income inequality were associated with lower levels of hsCRP. Importantly, we found that the variation in hsCRP associated with state-level socioeconomic conditions was clinically meaningful among low-income women. High-risk hsCRP values (≥ 3.0 mg/L) were seen among low-income women in the most deprived states, across almost all state-level indicators. State-level income inequality was correlated with all three biomarkers. In particular, decreasing state-level income inequality was associated with smaller variation in hsCRP between low-income and high-income women. Personal household income, however, appeared to be more strongly correlated with sICAM-1 and fibrinogen than state-level socioeconomic conditions.

Our study contributes to the public health literature on cardiovascular disease prevention by exploring the relation of macro-level socioeconomic conditions to risk factors for cardiovascular disease in healthy women. We find that both relative (inequality) and absolute (wealth and prosperity, productivity, poverty) measures of socioeconomic conditions at the state-level are associated with inflammation among women in excess of their personal income. To our knowledge, our study is the first to relate macro-level socioeconomic conditions to biomarkers of inflammation among healthy women in a multilevel context. Previous studies have documented an association between state-level socioeconomic conditions and other health outcomes, including modest associations between state-level income inequality and both mortality and self-rated health [32-36]. Our results are consistent with findings by Diez-Roux et al. who observed correlations between state-level income inequality and the presence of other cardiovascular disease risk factors, including increased BMI, high blood pressure, and sedentary behavior among women, where the largest effect sizes were observed in women with low-incomes [16].

The WHS is a cohort of predominantly white women employed in health professions, who have a low prevalence of traditional cardiovascular risk factors. Prior data from the WHS show that increased hsCRP, in particular, predicts incident myocardial infarction and stroke in this lower-risk population [4]. Our study generates the hypothesis that there are state-level socioeconomic pathways that impact inflammation, particularly hsCRP, among even healthy women in this demographic who have low-levels of traditional cardiovascular risk factors. Our study also raises the question of why macro-level socioeconomic conditions should matter to the health of healthy women. Previous studies of area-level contributors to inflammation address this question with a focus on neighborhood environments, and suggest that contextual differences are due to neighborhood conditions that promote psychosocial stress, influence social norms, or fail to facilitate healthy behaviors due to a lack of health-promoting resources [9,37,38]. In contrast, state-level socioeconomic conditions could potentially relate to health status among women through differences in state-level public health outlays, state investments in safety-net insurance that influence early health care seeking behavior, or by influencing the ability for states to contribute resources to local agencies that foster health promotion efforts [39-41]. Understanding state-level influences on these early biomarkers of atherosclerosis may be important to shape novel population-level prevention efforts in healthy women. Additional research is needed to generate theories on the impact of macro socioecomonic contexts on cardiovascular disease risk [39,42].

Important limitations of our study should be considered, including the cross-sectional design which precludes inferring any causal relationships between biomarkers of inflammation and state-level socioeconomic conditions. Additionally, our study focuses specifically on cardiovascular inflammation among a cohort of middle-aged and older women with a low prevalence of cardiovasular risk factors. A strength of this approach is that we are able to identify correlates of inflammation early in the course of atherosclerosis, where public health prevention may be most successful in slowing the progression of cardiovascular disease. The greatest limitation of this approach is the limit on generalizability to higher-risk cohorts, including cohorts with greater ethnic diversity and greater comorbidities. Our findings should be replicated in additional cohorts to assess their generalizability and robustness. A related limitation is that the WHS cohort was observed during an economic downturn in the 1990s. Additional work in other cohorts would be needed to observe the effect of state-level socioeconomic conditions in better national economic times, and to ascertain whether our findings represent a cohort-specific phenomenon. Last, we measure exposure to state conditions in mid to late adulthood, and cannot explore important relationships between inflammation and socioeconomic conditions experienced across the lifecourse, as has been observed at the individual-level in other studies [43].

## Conclusions

Our study advances knowledge of cardiovascular disease risk factors among healthy women by demonstrating a clinically meaningful change in cardiovascular inflammation associated with state-level socioeconomic conditions, particularly for hsCRP. The next steps of this line of investigation would include replicating our findings in other cohorts, and exploring mediating or confounding contextual influences that could explain these results. At this writing, the current macro socioeconomic environment presents multiple natural experiments at the state-level that will lead to state-level differences in resources devoted to health safety-nets and socioeconomic resources. Thus, attention to changes in cardiovascular risk factors associated with evolving state-level socioeconomic conditions may yield insights into the role of state-level policies in the prevention of cardiovascular disease among women [10,44]. If our findings are replicated, additional research should explore what more advantaged states do differently than less-advantaged states, that may contribute to reducing early biomarkers of atherosclerosis in their populations.

## Competing interests

Dr. Ridker is listed as a co-inventor on patents held by the Brigham and Women's Hospital that relate to the use of inflammatory biomarkers in cardiovascular disease that have been licensed to Siemens and AstraZeneca. The other authors report no conflicts to declare.

## Authors' contributions

CRC conceived of the study, acquired the data, analyzed and interpreted the data, drafted and revised the manuscript, performed the statistical analysis and obtained funding. PMR conceived of the study, acquired the data, analyzed and interpreted the data, revised the manuscript, obtained funding and provided study supervision. MJO and CEH analyzed and interpreted the data and drafted and revised the manuscript. BC conceived of the study, analyzed and interpreted the data, revised the manuscript, performed the statistical analysis and provided study supervision. JEB acquired the data, revised the manuscript and obtained funding. LFB conceived of the study, analyzed and interpreted the data, revised the manuscript and provided study supervision. All authors read and approved the final manuscript.

## Pre-publication history

The pre-publication history for this paper can be accessed here:

http://www.biomedcentral.com/1471-2458/12/211/prepub
